# Comparative Overall Survival of CDK4/6 Inhibitors Plus Endocrine Therapy vs. Endocrine Therapy Alone for Hormone receptor-positive, HER2-negative metastatic breast cancer

**DOI:** 10.7150/jca.48944

**Published:** 2020-10-18

**Authors:** Mingxi Lin, Yang Chen, Yizi Jin, Xichun Hu, Jian Zhang

**Affiliations:** Department of Medical Oncology, Fudan University Shanghai Cancer Center, Department of Oncology, Shanghai Medical College, Fudan University, Shanghai 200032, China

**Keywords:** Abemaciclib, Ribociclib, Palbociclib, Breast cancer, Meta-analysis

## Abstract

**Background**: The combination of CDK4/6 inhibitors and endocrine therapy has greatly improved progression-free survival (PFS) in patients with hormone receptor-positive, human epidermal growth factor receptor 2 (HER2)-negative metastatic breast cancer in many randomized controlled trials (RCTs). However, the key issue was the extent to which the benefit in PFS could translate into a prolongation of OS.

**Methods:** We performed a systematical literature search of PubMed, Web of Science, Cochrane Central Register of Clinical Trials and Embase, as well as meeting online archives up to February 2020. The primary outcome was OS, and we performed indirect treatment comparisons depend on a meta-analysis.

**Results:** Six RCTs were eligible including 3421 breast cancer patients. Compared to the endocrine therapy alone group, adding CDK4/6 inhibitors to endocrine therapy had significantly improved OS (HR=0.76, 95% CI=0.68-0.85, P<0.001). Moreover, the OS advantage was consistent in patients with different combined endocrine therapy, endocrine sensitivity status, sites of distant metastasis, menopausal status and age. Nevertheless, more adverse events were observed in patients treated with CDK4/6 inhibitors. The most common grade 3-4 adverse events were neutropenia (risk ratio [RR]=37.15, 95% CI=15.33-90.04), leucopenia (RR=25.58, 95% CI=13.23-49.46) and anaemia (RR=2.24, 95% CI=1.38-3.85).

**Conclusions:** Our meta-analysis suggested that compared with endocrine therapy alone, the addition of CDK4/6 inhibitors significantly improved OS in patients with hormone receptor-positive, HER2-negative metastatic breast cancer. However, the addition of CDK4/6 inhibitors also increased the incidences of grade 3-4 adverse events.

## Introduction

Breast cancer is one of the most common cancers and the leading cause of cancer-related deaths among women^1^. Hormone-receptor positive and human epidermal growth factor receptor 2 (HER2) negative breast cancer accounts for approximately 66% of all malignant neoplasms of the breast^2^-^4^. Most hormone-receptor positive breast cancer can be cured by adjuvant endocrine therapy in the early stage. However, there are still a small number of patients who went through recurrence and distant metastasis due to endocrine therapy resistance. Cyclin-dependent kinase 4/6 (CDK4/6) is a vital regulator of the cell cycle^5^. It accelerates the process of G1 phase and makes tumor cells proliferate rapidly through cyclin D-CDK 4/6-retinoblastoma pathway^6^. The dysregulated cyclin D-CDK4/6-retinoblastoma pathway is associated with endocrine therapy resistance^7^.

At present, three kinds of CDK4/6 inhibitors have achieved satisfactory results in clinical trials, including palbociclib^8^-^10^, ribociclib^11^-^14^ and abemaciclib^15^, ^16^. Several clinical trials have shown that the combination of CDK4/6 inhibitors and endocrine therapy can improve the progression free survival (PFS) compared to endocrine therapy alone. The benefit of the addition of palbociclib was studied in phase II clinical trial PALOMA-1^10^ in 2015. Compared with the letrozole alone group, the PFS was significantly improved in the combination group (20.2 vs 10.2 months, HR=0.488, 95% CI=0.319-0.748, P=0.0004). After PALOMA-1, ribociclib-based MONALEESA-2^14^, MONALEESA-3^12^, MONALEESA-7^13^ and palbociclib-based PALOMA-2^17^, PALOMA-3^8^ and abemaciclib-based MONARCH-2^15^, MONARCH-3^16^ all showed the superiority of CDK4/6 inhibitors-containing regimens over endocrine therapy alone. Based on the statistically significant and clinically meaningful improvements in PFS data, the US Food and Drug Administration (FDA) has approved CDK4/6 inhibitors in combination with endocrine therapy as first/second-line treatment in patients with hormone-receptor positive, HER2 negative metastatic breast cancer. However, the key issue was the extent to which this benefit in PFS could translate into a prolongation of OS.

In PALOMA-1, OS was not significantly prolonged (37.5 vs. 34.5 months, HR=0.897, 95% CI=0.623-1.294, P=0.281)^18^. However, in MONARCH-2, a phase 3 clinical trial, median OS was increased to 46.7 months in the abemaciclib plus fulvestrant group compared with 37.3 months for fulvestrant alone group (HR=0.757, 95% CI=0.606-0.945, P=0.01)^19^. Moreover, compared to placebo plus nonsteroidal aromatase inhibitor (NSAI)/tamoxifen regimen, the combination of ribociclib and NSAI/tamoxifen showed a significantly prolonged OS (not reached vs 40.9 months, HR=0.71, 95% CI=0.54-0.95, P=0.00973) as first-line or second-line treatment in the MONALEESA-7 trial^20^. The effect of CDK4/6 inhibitors on OS was not completely consistent in different clinical trials.

In this study, we performed indirect treatment comparisons depend on meta-analysis. We aimed to evaluate the OS benefit of adding CDK4/6 inhibitors to endocrine therapy in patients with hormone receptor-positive, HER2-negative metastatic breast cancer, as well as the patient subgroups that might benefit most from CDK4/6 inhibitors.

## Material and methods

### Literature search

We performed a systematical literature search to identify published phase II/III RCTs evaluating the clinical efficacy of endocrine therapy with or without CDK4/6 inhibitors in hormone receptor-positive, HER2-negative metastatic breast cancer. We searched the PubMed, Web of Science, Cochrane Central Register of Clinical Trials and Embase, as well as American Society of Clinical Oncology (ASCO) and San Antonio Breast Cancer Symposiums (SABCS) and European Society for Medical Oncology (ESMO) meeting online archives (up to February 2020). The detailed search strategy is provided in the supplementary (p1-p2).

### Eligibility criteria

To be included, studies should meet the following inclusion criteria based on PICOS principles:1) P(population): eligible patients were diagnosed with hormone receptor-positive, HER2-negative metastatic breast cancer. Studies enrolled patients with triple-negative or HER2-positive breast cancer were excluded; 2) I(intervention) and C(comparison): treatment with CDK4/6 inhibitor plus standard endocrine therapy in the experimental arm and endocrine therapy alone in the control arm; 3) O(outcome): overall survival (OS); 4) S(study design): phase II/III RCTs published in the form of full-text articles, or as abstracts if full-text articles were not available, were included. We excluded phase I trials, meta-analyses, reviews, preclinical studies, observational studies, single-arm-studies, non-randomized trials and subgroup analysis. No language restrictions were performed in our study. If several articles about the same clinical trial were identified, the most recent article was included.

### Data extraction

Two reviewers (CY and LM) independently reviewed and extracted the data, and a third reviewer (JYZ) was consulted to resolve the disagreement. The following information was extracted from the six included study: year of publication, phase of the trial, line of treatment, single-center or multi-center study, sample size, treatments for the intervention arm and control arm, hazard ratios (HR) and 95% confidence intervals (CI) of OS, median follow-up time, and information about the participants (menopausal status, the site of metastatic disease, age, endocrine sensitivity status, hormone-receptor status).

### Statistical analysis

The primary outcome was OS, which was defined as the time from randomization to date of death of any cause. HRs and the corresponding 95% CI were calculated for the effect of CDK4/6 inhibitor plus endocrine therapy versus endocrine therapy alone in terms of OS. The primary analysis was performed by all the included studies. The subgroup analyses were performed, and we preset the subgroups: 1) different CDK4/6 inhibitors (palbociclib vs abemaciclib vs ribociclib); 2) different endocrine therapy (aromatase inhibitor±CDK4/6 inhibitor vs fulvestrant±CDK4/6 inhibitor); 3) different menopausal status (peri-/premenopausal vs postmenopausal); 4) different site of metastasis (visceral vs nonvisceral); 5) different age (<65 years old vs ≥65 years old); 6) different hormone-receptor status (ER-positive and PgR-positive vs other); 7) different endocrine sensitivity status (endocrine sensitive vs endocrine resistant). The endocrine sensitive population is defined as patients with relapse interval > 12 months from completion of (neo)adjuvant endocrine therapy or patients have not received endocrine therapy. The endocrine resistant population is defined as patients relapsed during or within 12 months after completion of (neo)adjuvant endocrine therapy, or patients progressed on the first-line therapy. The endocrine resistant population could be divided into two population. One is the primary endocrine resistant population, and the other is the secondary endocrine resistant population. The primary endocrine resistance and secondary endocrine resistance are defined according to the 4^th^ ESO-ESMO International Consensus Guidelines^21^. Primary endocrine resistance is defined as relapse while on the first 2 years of adjuvant endocrine therapy, or progressed within first 6 months of first-line endocrine therapy. Secondary endocrine resistance is defined as relapse while on adjuvant endocrine therapy but after the first 2 years, or relapse within 12 months of completing adjuvant endocrine therapy, or progressed 6 months after initiating endocrine therapy for advanced breast cancer.

I^2^ statistics were used to evaluate the heterogeneity among the included studies. If I^2^≥50% and/or P value<0.10, the heterogeneity was considered statistically significant, and a random effect model was performed to pool the HRs. Otherwise, a fixed effects model was performed^22^. For the publication bias assessment, Begg's and Egger's tests as well as the funnel plot were performed^23^, ^24^. The risk of bias assessment of the included randomized controlled trials was performed using the Cochrane Collaboration's Tool^25^.

All statistical analyses were performed using Stata (Version 15.0). All statistical tests were two-tailed and p<0.05 was considered statistically significant.

## Results

### Study selection and the associated characteristic

**Figure 1:**
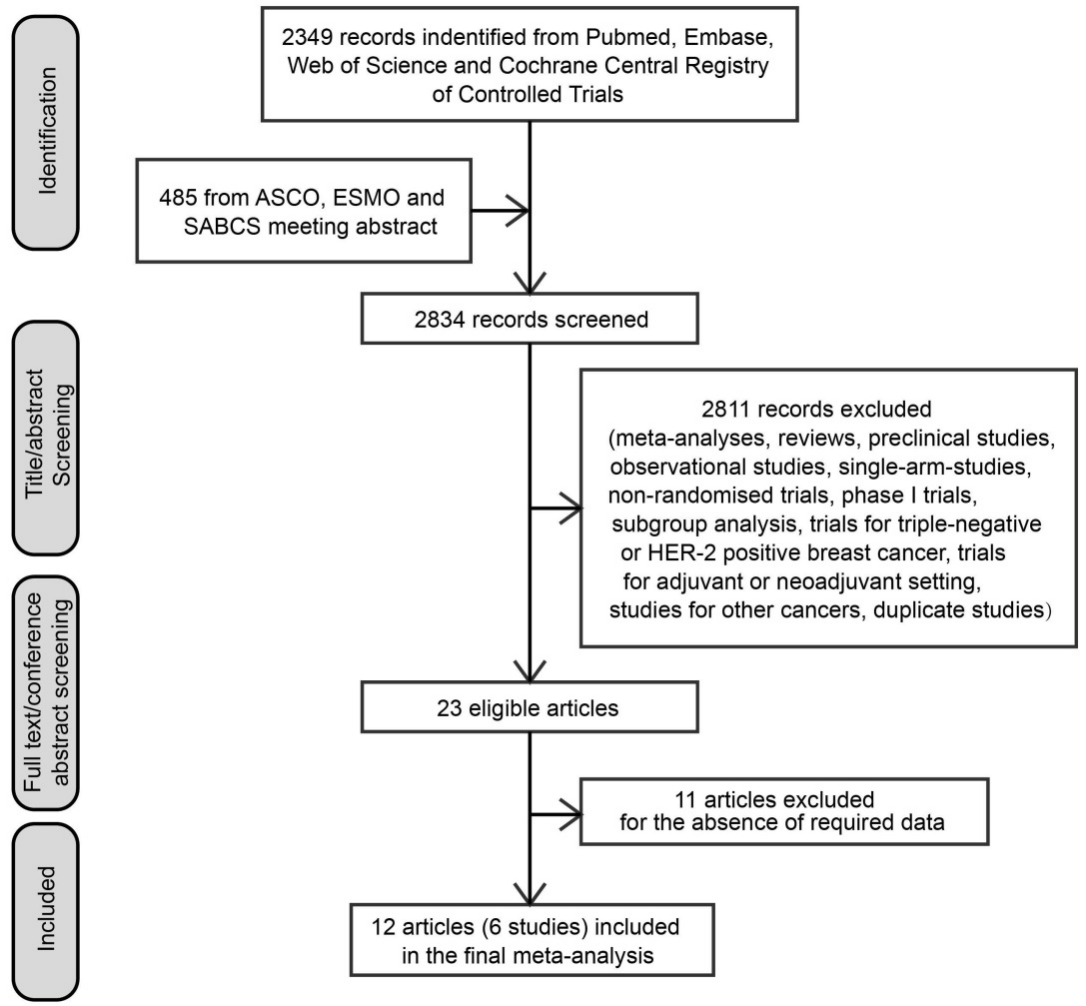
Flow-chart of literature search. Abbreviations: ASCO, American Society of Clinical Oncology; ESMO, European Society of Medical Oncology; SABCS, San Antonio Breast Cancer Symposiums.

A total of 2834 articles were first identified from literature screening. According to our eligibility criteria, 2811 articles were excluded after title/abstract reviewing, and 11 articles were excluded after full-text reviewing. Finally, six trials were included in our meta-analysis (Figure 1) 11, 18-20, 26, 27.

In the six included RCTs, a total of 3421 patients [median with range: 668.5 (165-726)] were enrolled. Five (83%) of the six trials were phase III studies, and only one (17%) trial was a phase II study. All the six trials were multicenter studies. One (17%) RCT was first-line treatment, two (33%) RCTs were second-line or later treatment, and three (50%) RCTs included both first-line and second-line treatments. All the six trials compared the standard endocrine therapy plus CDK4/6 inhibitor with endocrine therapy alone, but the CDK4/6 inhibitors used were different. One (17%) used abemaciclib, two (33%) used palbociclib and three (50%) used ribociclib. Three (50%) of the six trials used aromatase inhibitor as standard endocrine therapy in the control arm, while the other three (50%) used fulvestrant. A detailed description of the characteristics of the included studies is presented in Table 1.

### CDK4/6 inhibitor use and overall survival (OS)

**Figure 2:**
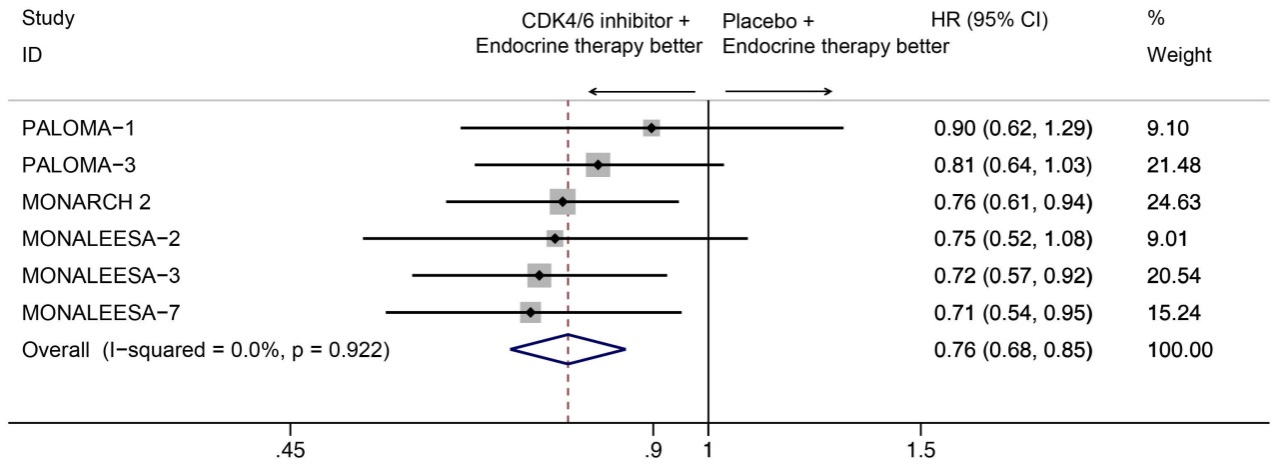
Forest plot showing pooled hazard ratios of overall survival for ET plus CDK4/6 inhibitor vs ET alone

Our results indicated that CDK4/6 inhibitor use was positively associated with OS (HR=0.76, 95% CI=0.68-0.85, P<0.001; Figure 2). And there was no statistically significant heterogeneity among the six studies (I^2^=0.0%, P=0.922), suggesting that the OS advantage was consistent among the studies.

**Figure 3:**
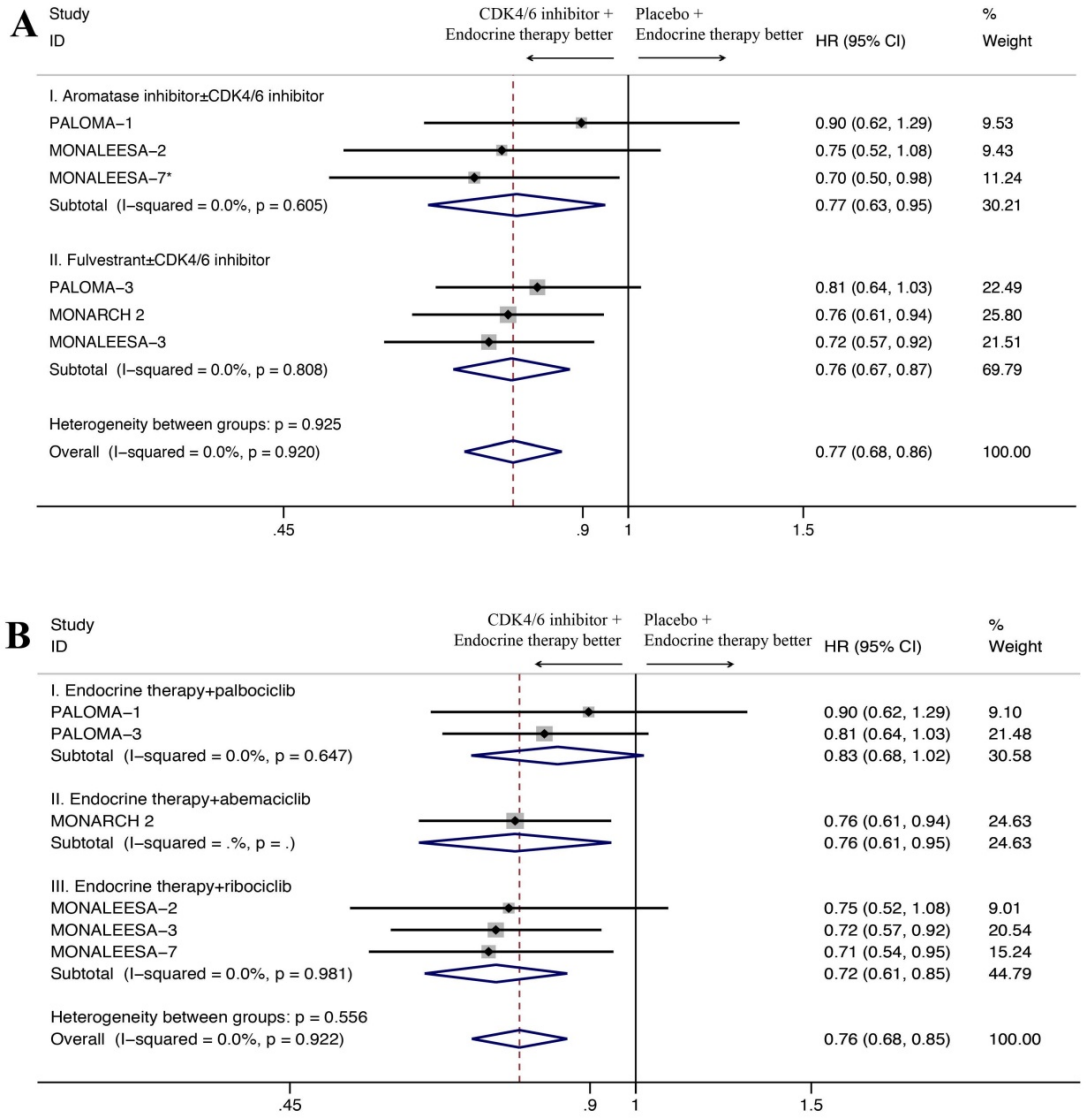
Forest plot showing pooled hazard ratios of overall survival in subgroups stratified by combined endocrine therapy (A) and different CDK4/6 inhibitors (B). *Only 495 of 672 patients received aromatase inhibitor as combined endocrine therapy were included.

For subgroup analysis, CDK4/6 inhibitors combined with an aromatase inhibitor had a favorable impact on OS compared with aromatase inhibitor alone without heterogeneity (HR=0.77, 95% CI=0.63-0.95, P=0.014, I^2^=0.0%; Figure 3A), and the similar results were observed for patients receiving CDK4/6 inhibitor plus fulvestrant (HR=0.76, 95% CI=0.67-0.87, P<0.001, I^2^=0.0%). Moreover, subgroup analysis was done among patients treated with different CDK4/6 inhibitors. Three trials have ribociclib-based regimen. As seen in Figure 3B, the pooled analysis showed statistically significant better OS among patients treated with ribociclib plus endocrine therapy (HR=0.72, 95% CI= 0.61-0.85, P<0.001). With an I^2^ of 0, the results of the three trials showed no heterogeneity. For the two trials with a palbociclib-based regimen, palbociclib plus endocrine therapy showed no significantly better OS than endocrine therapy alone (HR=0.83, 95% CI=0.68-1.02, P=0.076, I^2^=0.0%). Only one trial used the abemaciclib-based regimen, and also showed better OS with the addition of CDK4/6 inhibitors (HR=0.76, 95% CI=0.61-0.95, P=0.014).

**Figure 4:**
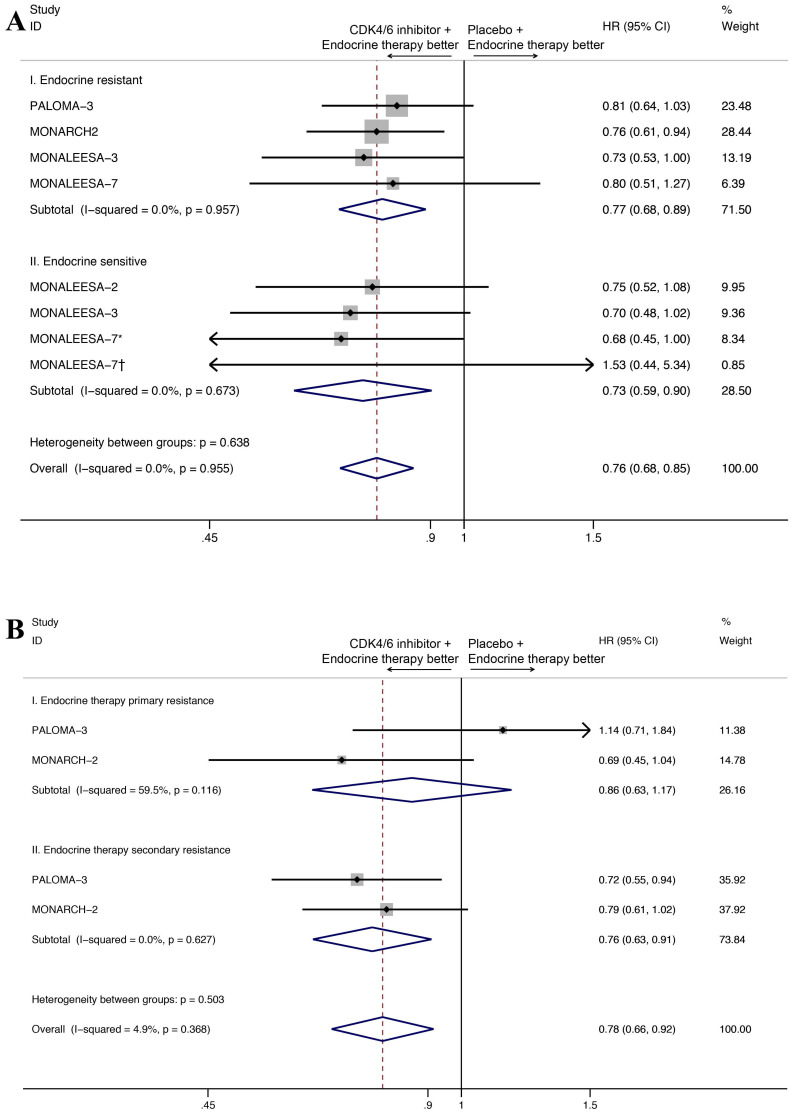
Forest plot showing pooled hazard ratios of overall survival in subgroup stratified by combined endocrine sensitivity status (A), primary/secondary resistance(B). *Patients with no previous endocrine therapy. †Patients with relapse interval > 12 months from completion of (neo)adjuvant endocrine therapy

As for the endocrine sensitivity status, four studies provided the OS results for the endocrine resistant subset and three studies provided the OS results for endocrine sensitive subset (Figure 4A). The CDK4/6 inhibitors combined with endocrine therapy had a favorable impact on OS in endocrine resistant subset (HR=0.77, 95%CI=0.68-0.89, P<0.001, I^2^=0.0%), as well as the endocrine sensitive subset (HR=0.73, 95%CI=0.59-0.90, P=0.004, I^2^=0.0%). Moreover, two studies reported OS results for endocrine therapy primary resistance subset and endocrine therapy secondary resistance subset (Figure 4B). Our results indicated that the addition of CDK4/6 inhibitors was positively associated with OS in the latter group (HR=0.76, 95% CI=0.63-0.91, P=0.003, I^2^=0.0%), but not for the first group (HR=0.86, 95% CI=0.63-1.17, P=0.330, I^2^=59.5%).

We also analyzed overall survival in exploratory subgroups, including menopausal status, the site of metastatic disease, age, hormone-receptor status (Table 3, Figure S1). In general, the advantage of CDK4/6 inhibitor use was consistent with that observed in the overall population. However, the results of some subgroup analysis showed no significant difference, owing to the small number of patients included.

### CDK4/6 inhibitor use and adverse events

We performed an analysis of the top 10 adverse events (neutropenia, nausea, fatigue, diarrhoea, arthralgia, leucopenia, headache, vomiting, hot flush, anaemia) between the two groups. In terms of all grade adverse events, CDK4/6 inhibitors plus endocrine therapy group showed significantly higher rates of neutropenia (risk ratio [RR]=14.77, 95% CI=10.26-21.26), nausea (RR=1.66,95% CI=1.49-1.85), fatigue (RR=1.22, 95% CI=1.02-1.45), diarrhoea (RR=1.64, 95% CI=1.08-2.48), leucopenia (RR=9.95, 95% CI=7.43-13.32), vomiting (RR=1.74, 95% CI=1.29-2.34), anaemia (RR=3.53, 95% CI=2.36-5.26) (Table 3).

In terms of grade 3-4 adverse events, neutropenia and leucopenia are the most commonly observed adverse events with an RR 37.15 (95% CI=15.33-90.04) for neutropenia and 25.58 (95% CI=13.23-49.46) for leucopenia. Febrile neutropenia occurred in only 1.14% of patients in CDK4/6 inhibitors plus endocrine therapy group and 0.20% of patients in endocrine therapy alone group. In the subgroup analysis of different CDK4/6 inhibitors (Figure S2), the incidences of grade 3-4 neutropenia were significantly higher in patients receiving ribociclib-based regimen (RR=47.33, 95% CI=9.67-231.61), and palbociclib-based regimen (RR=68.15, 95%CI=17.09-271.83). Moreover, patients received abemaciclib-based regimen showed higher rates of grade 3-4 diarrhoea.

### The risk of bias and publication bias assessment

The risk of bias assessment of the included RCTs are summarized in sTable 1 using the Cochrane Collaboration's Tool 25. The PALOMA-1 was an open-label trial with a high risk of inadequate blinding. However, the other included trials were all phase III, double-blinded trials with low risk of bias for most assessments.

**Figure 5:**
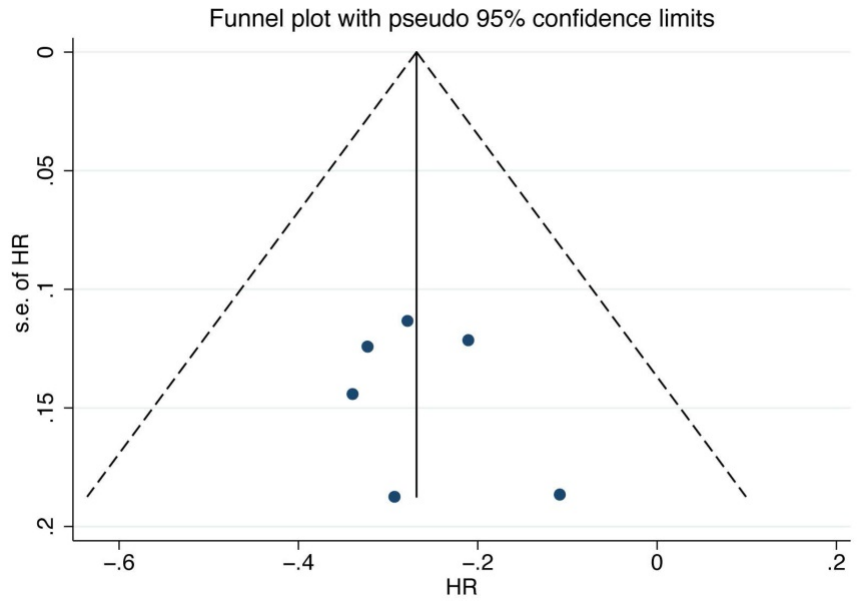
Funnel plot of the overall survival

For the assessment of publication bias, a funnel plot was performed, and the plot showed mild asymmetry (Figure 5). The results of Begg's and Egger's tests (P_Begg's_=1.000, P_Egger's_=0.553) also indicated that there was no significant publication bias.

## Discussion

To our knowledge, this is the first comprehensive meta-analysis including all the published OS data to assess the OS benefit of CDK4/6 inhibitors. Pivotal phase III clinical trials all suggested that the addition of CDK4/6 inhibitors to endocrine therapy can greatly improve PFS in patients with hormone receptor-positive, HER2-negative metastatic breast cancer 8, 10-13, 15. However, OS data were not mature for many of the studies at the time of PFS analysis. Until recently, OS data were only available for PALOMA 1^18^, PALOMA 3^26^ and MONALEESA 2^11^. Two previous meta-analyses^28^, ^29^ were not powered to detect an overall survival advantage, for the reason that the majority of survival results of the RCTs included were still pending. For the US Food and Drug Administration pooled analysis^30^, a non-statistically significant OS benefit was observed across all the pooled trials (0.89, 95% CI 0.78-1.01). Because the efficacy data were extracted on April 30, 2018, and overall survival data were not mature for all the pooled studies at that time. Li et al^31^ performed a meta-analysis and firstly detected the OS benefit of CDK4/6 inhibitors (HR 0.79, 95% CI 0.67-0.93), however they only included 3 RCTs. The meta-analysis performed by Schettini et al^32^ also detected the OS benefit of CDK4/6 inhibitors, but they did not include the updated OS data of PALOMA-1 published in 2017^18^. Recently, OS data for pivotal phase III trials MONARCH2, MONALEESA 3 and MONALEESA 6 were published. Our meta-analysis carefully included all the six randomized clinical trials and the updated OS results. We showed that CDK4/6 inhibitors plus endocrine therapy, compared with endocrine therapy alone, significantly improved OS in patients with hormone receptor-positive, HER2-negative metastatic breast cancer. Our study detected an OS advantage among these six clinical trials, regardless of the combined endocrine therapy, endocrine sensitivity status, sites of distant metastasis, menopausal status and age. Some OS data of other clinical trials are still pending, such as PALOMA-2 and MONARCH 3.

The subgroup analysis of different CDK4/6 inhibitors showed that palbociclib did not significantly improve OS in patients, and this result was not consistent with the significant improvements in PFS observed in the two palbociclib-based clinical trials (PALOMA-1 and PALOMA-3). We speculate that the inconsistency between OS and PFS may be due to the different subsequent treatment regimens and the cross-over from the control arm to CDK4/6 inhibitors treatment. In this situation, the benefit in PFS could not translate to a prolongation of OS. Moreover, even if the analysis of OS did not meet the threshold for statistical significance, the addition of CDK4/6 inhibitors to endocrine therapy resulted in a prolongation of OS of 6.9 months in PALOMA-3, and 3.0 months in PALOMA-1.

The OS benefits of CDK4/6 inhibitors were observed in both endocrine sensitive subgroup (HR=0.73, 95% CI=0.59-0.90, P=0.004) and endocrine resistant subgroup (HR=0.77, 95% CI=0.68-0.89, P<0.001), which was consistent with the findings of the preclinical studies. Preclinical studies showed that cyclin-dependent kinases (CDKs) played an essential role in regulating cell-cycle progression^33^. Alterations in the cyclin-D-CDK4/6-retinoblastoma pathway were associated with endocrine resistance in breast cancer, and the CDK4/6 inhibitors had shown its ability to reverse endocrine resistance^34^-^36^. A meta-analysis also showed that adding CDK4/6 inhibitors in endocrine-sensitive (HR 0.55, 95% CI 0.50-0.62) or endocrine-resistant setting (HR 0.51, 95% CI 0.43-0.61) significantly improved the PFS of metastatic hormone receptor-positive, HER2-negative breast cancers regardless of menopausal status and site of metastasis^29^. However, when we analyzed the patients with primary endocrine resistance and the patients with secondary endocrine resistance separately, the OS benefit of CDK4/6 inhibitors was only observed in the latter group. In MONARCH 2, OS subgroup analysis done in patients with primary vs secondary endocrine resistance showed a better OS effect in patients with primary endocrine resistance (HR 0.69, 95% CI 0.45-1.04)^19^. This contrasted with the OS data in the PALOMA 3 study. A subgroup analysis in PALOMA 3 indicated no better OS effect in patients with primary endocrine resistance (HR 1.14, 95% CI 0.71-1.84)^26^. The divergent results suggested a potential differential activity between abemaciclib and palbociclib in patients with primary endocrine resistance. Further studies are warranted to draw more definitive conclusions.

In this study, there were also some unavoidable problems in the process of analysis. The methods for subgroup stratifications were not precisely the same. For example, in MONALEESA-3 and MONALEESA-7 clinical trials, visceral metastases only referred to lung and/or liver metastases, while in MONARCH-2 and PALOMA-3 clinical trials, visceral metastases referred to lung, liver, brain, pleural, and peritoneal involvement^12^, ^19^, ^20^, ^26^. Therefore, bias might exist in the subgroup analysis of visceral metastasis subset vs non-visceral metastasis subset. However, for patients with metastatic hormone receptor-positive breast cancer, visceral metastases mainly referred to lung and/or liver metastases 37. Metastatic hormone receptor-positive breast cancer had a high incidence of metastasis to liver (28.6% for luminal A, 32.0% for luminal B) and lung (23.8% for luminal A, 30.4% for luminal B). Low risks of brain metastases (2.2% with luminal A and 4.7% with luminal B) were seen. Based on this, the bias will not have a severe impact on the final results of the analysis^38^.

Adding CDK4/6 inhibitors increased the rate of 3-4 adverse events. The most significant adverse events are mainly related to the blood system, such as neutropenia (RR=37.15, 95% CI=15.33-90.04), leucopenia (RR=25.58, 95% CI=13.23-49.46) and anaemia (RR=2.24, 95% CI=1.38-3.85). There was no significant difference in the incidence of grade 3 digestive system adverse events such as diarrhea and vomiting. It suggested that we need to closely monitor the hemogram of patients to prevent or deal with serious adverse events in time when using CDK4/6 inhibitors.

## Conclusion

Our meta-analysis indicated that compared with endocrine therapy alone, the addition of CDK4/6 inhibitors significantly improved OS in patients with hormone receptor-positive, HER2-negative metastatic breast cancer. The advantage of CDK4/6 inhibitor was consistent in patients with different combined endocrine therapy, endocrine sensitivity status, sites of distant metastasis, menopausal status and age. However, the addition of CDK4/6 inhibitors also associated with a higher rate of grade 3-4 adverse events.

## Supplementary Material

Supplementary material, figures, table.

## Figures and Tables

**Table 1:** Characteristics of studies included in the meta-analysis

Study	Line*	Phase	Centers	Patient characteristics	Arm A	Arm B	Sample size	Median follow-up time	OS
PALOMA-1^10^, ^18^	First-line or second-line	II	Multicenter	Postmenopausal women	Palbociclib + letrozole	Letrozole	165 (84:81)	Not reported	37.5 months vs 34.5 months, HR 0.897 (95%CI 0.623-1.294)
PALOMA-3^8^, ^26^	Second-line or later	III	Multicenter	Any menopausal status	Fulvestrant + palbociclib	Fulvestrant + placebo	521 (347:174)	44.8 months	34.9 months vs 28.0 months, HR 0.81 (95%CI 0.64-1.03)
MONARCH-2^15^, ^19^	Second-line	III	Multicenter	Any menopausal status	Abemaciclib + fulvestrant	Placebo + fulvestrant	669 (446:223)	47.7 months	46.7 months vs 37.3 months, HR 0.757 (95%CI 0.606-0.945)
MONALEESA-2^11^, ^14^	First-line	III	Multicenter	Postmenopausal women	Ribociclib + letrozole	Placebo + letrozole	668 (334:334)	26.4 months	Immature, HR 0.746 (95%CI 0.517-1.078)
MONALEESA-3^12^, ^27^	First-line or second-line	III	Multicenter	Men and postmenopausal women	Ribociclib+ fulvestrant	Placebo+ fulvestrant	726 (484:242)	29.4 months	Not reached vs 40.0 months, HR 0.72 (95%CI 0.57-0.92)
MONALEESA-7^12^, ^20^	First-line or second-line	III	Multicenter	Pre- or perimenopause	Ribociclib+ NSAI/tamoxifen	Placebo + NSAI/tamoxifen	672 (335:337)	34.6 months	Not reached vs 40.9 months, HR 0.71 (95%CI 0.54-0.95)

Abbreviations: HR, Hazard ratio; CI, Confidence interval; OS, Overall survival; NSAI, Nonsteroidal aromatase inhibitor * First-line therapy is defined as newly diagnosed advanced disease with no systemic therapy in the metastatic setting and relapse > 12 months from completion of (neo)adjuvant endocrine therapy. Second-line therapy is defined as relapse during or within 12 months after completion of (neo)adjuvant endocrine therapy and disease progressed after one line of therapy for advanced disease

**Table 2:** Subgroup analyses according to clinicopathological characteristics

Subgroup	Pooled HR	95% CI	Heterogeneity I^2^; P-value	P-value for heterogeneity between subgroups
Menopausal status				0.873
Peri-/premenopausal	0.76	0.60-0.96	0.0%; 0.416	
Postmenopausal	0.74	0.64-0.86	0.0%; 0.920	
Site of metastatic disease				0.620
Visceral	0.76	0.65-0.89	0.0%; 0.686	
Nonvisceral	0.71	0.58-0.88	0.0%; 0.736	
Age				0.487
<65 yr	0.80	0.67-0.95	0.0%; 0.445	
≥65 yr	0.72	0.58-0.90	44.4%; 0.166	
Hormone-receptor status				0.872
ER-positive and PgR-positive	0.75	0.63-0.89	0.0%; 0.967	
Other	0.77	0.58-1.01	0.0%; 0.743	

Abbreviations: HR, Hazard ratio; CI, Confidence interval; ER, Estrogen receptor; PgR, Progesterone receptor

**Table 3:** Top 10 adverse events associated with CDK4/6 inhibitors plus endocrine therapy versus endocrine therapy alone

Adverse effects	CDK4/6 inhibitors +ET group (event/total)	Placebo + ET group (event/total)	Total incidence (%)	RR (95%CI)	P value	Heterogeneity	
						I^2^ (%)	P value
All grade							
Neutropenia	1382/2021	67/1380	42.61	14.77 (10.26-21.26)	<0.001	50.4	0.073
Nausea	829/2021	336/1380	34.25	1.66 (1.49-1.85)	<0.001	36.8	0.162
Fatigue	698/2021	389/1380	31.96	1.22 (1.02-1.45)	0.029	62.3	0.021
Diarrhoea	797/2021	280/1380	31.67	1.64 (1.08-2.48)	0.020	90.3	<0.001
Arthralgia	426/2021	322/1380	21.99	0.97 (0.86-1.10)	0.649	0.0	0.593
Leucopenia	684/2021	49/1380	21.55	9.95 (7.43-13.32)	<0.001	46.0	0.099
Headache	436/2021	269/1380	20.73	1.12 (0.97-1.28)	0.111	0	0.709
Vomiting	475/2021	189/1380	19.52	1.74 (1.29-2.34)	<0.001	67.3	0.009
Hot flush	364/2021	292/1380	19.29	0.96 (0.83-1.10)	0.522	0.0	0.470
Anaemia	468/2021	94/1380	16.52	3.53 (2.36-5.26)	<0.001	67.9	0.008
Grade 3-4							
Neutropenia	1044/2021	21/1380	31.31	37.15 (15.33-90.04)	<0.001	65.1	0.014
Leucopenia	336/2021	8/1380	10.11	25.58 (13.23-49.46)	<0.001	0.0	0.713
Anaemia	76/2021	22/1380	2.88	2.24 (1.38-3.85)	0.001	24.6	0.250
Diarrhoea	74/2021	8/1380	2.41	2.51 (0.55-11.42)	0.235	67.2	0.009
Fatigue	44/2021	8/1380	1.53	3.54 (1.70-7.40)	0.001	0.0	0.923
Nausea	31/2021	9/1380	1.18	2.19 (1.07-4.49)	0.032	0.0	0.647
Vomiting	28/2021	11/1380	1.15	1.75 (0.92-3.32)	0.087	45.3	0.104
Arthralgia	12/2021	10/1380	0.65	0.93 (0.40-2.14)	0.866	0.0	0.978
Headache	10/2021	6/1380	0.47	1.00 (0.36-2.78)	0.997	0.0	0.642
Hot flush	2/2021	1/1380	0.09	1.11 (0.25-4.91)	0.892	4.4	0.351

Abbreviations: ET, Endocrine Therapy; HR, Hazard ratio; RR, risk ratios; CI, Confidence interval; ER, Estrogen receptor; PgR, Progesterone receptor

## References

[1] Siegel RL, Miller KD, Jemal A (2019). Cancer statistics, 2019. CA: a cancer journal for clinicians.

[2] DeSantis CE, Ma J, Gaudet MM, Newman LA, Miller KD, Sauer AG (2019). Breast cancer statistics, 2019. CA-Cancer J Clin.

[3] Arpino G, Milano M, De Placido S (2015). Features of aggressive breast cancer. Breast.

[4] Perou CM, Sorlie T, Eisen MB, van de Rijn M, Jeffrey SS, Rees CA (2000). Molecular portraits of human breast tumours. Nature.

[5] Dempsey JA, Chan EM, Burke TF, Beckmann RP (2013). LY2835219, a selective inhibitor of CDK4 and CDK6, inhibits growth in preclinical models of human cancer. Cancer Res.

[6] VanArsdale T, Boshoff C, Arndt KT, Abraham RT (2015). Molecular Pathways: Targeting the Cyclin D-CDK4/6 Axis for Cancer Treatment. Clin Cancer Res.

[7] Thangavel C, Dean JL, Ertel A, Knudsen KE, Aldaz CM, Witkiewicz AK (2011). Therapeutically activating RB: reestablishing cell cycle control in endocrine therapy-resistant breast cancer. Endocrine-related cancer.

[8] Cristofanilli M, Turner NC, Bondarenko I (2016). Fulvestrant plus palbociclib versus fulvestrant plus placebo for treatment of hormone-receptor-positive, HER2-negative metastatic breast cancer that progressed on previous endocrine therapy (PALOMA-3): final analysis of the multicentre, double-blind, phase 3 randomised controlled trial (vol 17, pg 431, 2016). Lancet Oncology.

[9] Finn RS, Martin M, Rugo HS, Jones S, Im S-A, Gelmon K (2016). Palbociclib and Letrozole in Advanced Breast Cancer. New England Journal of Medicine.

[10] Finn RS, Crown JP, Lang I, Boer K, Bondarenko IM, Kulyk SO (2015). The cyclin-dependent kinase 4/6 inhibitor palbociclib in combination with letrozole versus letrozole alone as first-line treatment of oestrogen receptor-positive, HER2-negative, advanced breast cancer (PALOMA-1/TRIO-18): a randomised phase 2 study. The lancet Oncology.

[11] Hortobagyi GN, Stemmer SM, Burris HA, Yap YS, Sonke CS, Paluch-Shimon S (2018). Updated results from MONALEESA-2, a phase III trial of first-line ribociclib plus letrozole versus placebo plus letrozole in hormone receptor-positive, HER2-negative advanced breast cancer. Annals of Oncology.

[12] Slamon DJ, Neven P, Chia S, Fasching PA, De Laurentiis M, Im S-A (2018). Phase III Randomized Study of Ribociclib and Fulvestrant in Hormone Receptor-Positive, Human Epidermal Growth Factor Receptor 2-Negative Advanced Breast Cancer: MONALEESA-3. Journal of Clinical Oncology.

[13] Tripathy D, Im S-A, Colleoni M, Franke F, Bardia A, Harbeck N (2018). Ribociclib plus endocrine therapy for premenopausal women with hormone-receptor-positive, advanced breast cancer (MONALEESA-7): a randomised phase 3 trial. Lancet Oncology.

[14] Hortobagyi GN, Stemmer SM, Burris HA, Yap YS, Sonke GS, Paluch-Shimon S (2016). Ribociclib as First-Line Therapy for HR-Positive, Advanced Breast Cancer. New England journal of medicine.

[15] Sledge GW Jr, Toi M, Neven P, Sohn J, Inoue K, Pivot X (2017). MONARCH 2: Abemaciclib in Combination With Fulvestrant in Women With HR+/HER2-Advanced Breast Cancer Who Had Progressed While Receiving Endocrine Therapy. Journal of Clinical Oncology.

[16] Goetz MP, Toi M, Campone M, Sohn J, Paluch-Shimon S, Huober J (2017). MONARCH 3: Abemaciclib As Initial Therapy for Advanced Breast Cancer. Journal of Clinical Oncology.

[17] Finn RS, Martin M, Rugo HS, Jones S, Im SA, Gelmon K (2016). Palbociclib and Letrozole in Advanced Breast Cancer. New England journal of medicine.

[18] Finn RS, Crown J, Lang I, Boer K, Bondarenko I, Kulyk SO (2017). Overall survival results from the randomized phase II study of palbociclib (P) in combination with letrozole (L) vs letrozole alone for frontline treatment of ER+/HER2-advanced breast cancer (PALOMA-1; TRIO-18). Journal of Clinical Oncology.

[19] Sledge GW, Toi M, Neven P, Sohn J, Inoue K, Pivot X (2020). The Effect of Abemaciclib Plus Fulvestrant on Overall Survival in Hormone Receptor-Positive, ERBB2-Negative Breast Cancer That Progressed on Endocrine Therapy - MONARCH 2: A Randomized Clinical Trial. JAMA Oncology.

[20] Im SA, Lu YS, Bardia A, Harbeck N, Colleoni M, Franke F (2019). Overall Survival with Ribociclib plus Endocrine Therapy in Breast Cancer. New England journal of medicine.

[21] Cardoso F, Senkus E, Costa A, Papadopoulos E, Aapro M, Andre F (2018). 4th ESO-ESMO International Consensus Guidelines for Advanced Breast Cancer (ABC 4). Annals of Oncology.

[22] Schmidt FL, Oh IS, Hayes TL (2009). Fixed- versus random-effects models in meta-analysis: model properties and an empirical comparison of differences in results. The British journal of mathematical and statistical psychology.

[23] Egger M, Davey Smith G, Schneider M, Minder C (1997). Bias in meta-analysis detected by a simple, graphical test. BMJ (Clinical research ed).

[24] Begg CB, Mazumdar M (1994). Operating characteristics of a rank correlation test for publication bias. Biometrics.

[25] Higgins J GSe. Cochrane Handbook for Systematic Reviews of Interventions Version 5.2.0.; 2017

[26] Turner NC, Slamon DJ, Ro J, Bondarenko I, Im SA, Masuda N (2018). Overall Survival with Palbociclib and Fulvestrant in Advanced Breast Cancer. New England journal of medicine.

[27] Slamon DJ, Neven P, Chia S, Fasching PA, De Laurentiis M, Im S-A (2020). Overall Survival with Ribociclib plus Fulvestrant in Advanced Breast Cancer. The New England journal of medicine.

[28] Deng Y, Ma G, Li W, Wang T, Zhao Y, Wu Q (2018). CDK4/6 Inhibitors in Combination With Hormone Therapy for HR(+)/HER2(-) Advanced Breast Cancer: A Systematic Review and Meta-analysis of Randomized Controlled Trials. Clin Breast Cancer.

[29] Messina C, Cattrini C, Buzzatti G, Cerbone L, Zanardi E, Messina M (2018). CDK4/6 inhibitors in advanced hormone receptor-positive/HER2-negative breast cancer: a systematic review and meta-analysis of randomized trials. Breast Cancer Res Treat.

[30] Gao JJ, Cheng J, Bloomquist E, Sanchez J, Wedam SB, Singh H (2020). CDK4/6 inhibitor treatment for patients with hormone receptor-positive, HER2-negative, advanced or metastatic breast cancer: a US Food and Drug Administration pooled analysis. The Lancet Oncology.

[31] Li J, Fu F, Yu L, Huang M, Lin Y, Mei Q (2020). Cyclin-dependent kinase 4 and 6 inhibitors in hormone receptor-positive, human epidermal growth factor receptor-2 negative advanced breast cancer: a meta-analysis of randomized clinical trials. Breast Cancer Research and Treatment.

[32] Schettini F, Giudici F, Giuliano M, Cristofanilli M, Arpino G, Del Mastro L (2020). Overall survival of CDK4/6-inhibitors-based treatments in clinically relevant subgroups of metastatic breast cancer: systematic review and meta-analysis. J Natl Cancer Inst.

[33] Lukas J, Bartkova J, Bartek J (1996). Convergence of mitogenic signalling cascades from diverse classes of receptors at the cyclin D-cyclin-dependent kinase-pRb-controlled G1 checkpoint. Molecular and cellular biology.

[34] Finn RS, Dering J, Conklin D, Kalous O, Cohen DJ, Desai AJ (2009). PD 0332991, a selective cyclin D kinase 4/6 inhibitor, preferentially inhibits proliferation of luminal estrogen receptor-positive human breast cancer cell lines in vitro. Breast cancer research: BCR.

[35] Patnaik A, Rosen LS, Tolaney SM, Tolcher AW, Goldman JW, Gandhi L (2016). Efficacy and Safety of Abemaciclib, an Inhibitor of CDK4 and CDK6, for Patients with Breast Cancer, Non-Small Cell Lung Cancer, and Other Solid Tumors. Cancer Discov.

[36] Vidula N, Rugo HS (2016). Cyclin-Dependent Kinase 4/6 Inhibitors for the Treatment of Breast Cancer: A Review of Preclinical and Clinical Data. Clinical Breast Cancer.

[37] Minn AJ, Kang Y, Serganova I, Gupta GP, Giri DD, Doubrovin M (2005). Distinct organ-specific metastatic potential of individual breast cancer cells and primary tumors. The Journal of clinical investigation.

[38] Kennecke H, Yerushalmi R, Woods R, Cheang MC, Voduc D, Speers CH (2010). Metastatic behavior of breast cancer subtypes. Journal of clinical oncology: official journal of the American Society of Clinical Oncology.

