# Supplementary material for: Comparative Overall Survival of CDK4/6 Inhibitors Plus Endocrine Therapy vs. Endocrine Therapy Alone for Hormone receptor-positive, HER2-negative metastatic breast cancer
Source: J Cancer. 2020 Oct 18;11(24):7127–36. doi: 10.7150/jca.48944 (PMC7646186; doi:10.7150/jca.48944)
Supplement: Supplementary file 1 — Supplementary material, figures, table. [file jcav11p7127s1.pdf]

PUBMED

#1 breast cancer [MeSH Terms]

#2 (((breast OR mammary) AND (carcinoma OR neoplasm OR tumor OR cancer))

#3 #1 OR #2

#4 metastatic OR advanced OR metastases OR metastasis

#5 #3 AND #4

#6 'cyclin-dependent kinase 4 and 6 inhibitor*' OR 'cyclin-dependent kinase 4/6 inhibitor*' OR 'CDK4 and CDK6 inhibitor*' OR 'CDK4/6 inhibitor*' OR 'CDK inhibitor*' OR 'CDKi'

#7 palbociclib OR ribociclib OR abemaciclib

#8 #6 OR #7

#9 #5 AND #8

#10 ((((randomized controlled trial [pt] OR controlled clinical trial [pt] OR randomized controlled trials [mh] OR random allocation [mh] OR double-blind method [mh] OR single-blind method [mh] OR clinical trial [pt] OR clinical trials [mh] OR (「clinical trial」 [tw]) OR ((singl* [tw] OR doubl* [tw] OR trebl* [tw] OR tripl* [tw]) AND(mask* [tw] OR blind* [tw])) OR (placebos [mh] OR placebo* [tw] OR random* [tw] OR research design [mh:noexp])NOT (animals [mh] NOT human [mh])))))

#11 #9 AND #10

COCHRANE

#1 MeSH descriptor: [Breast Neoplasms] explode all trees

#2 metastatic OR metastasis OR metastases OR advanced

#3 #1 AND #2

#4 palbociclib OR ribociclib OR abemaciclib

#5 'cyclin-dependent kinase 4 and 6 inhibitor*' OR 'cyclin-dependent kinase 4/6 inhibitor*' OR 'CDK4 and CDK6 inhibitor*' OR 'CDK4/6 inhibitor*' OR 'CDK inhibitor*' OR 'CDKi'

#6 #4 OR #5

#7 #3 AND #6

EMBASE

#1 ('breast'/exp OR breast) AND ('cancer'/exp OR cancer)

#2 'metastatic':ab,ti OR 'metastasis':ab,ti OR 'metastases':ab,ti OR 'advanced':ab,ti

#3 #1 AND #2

#4 'cyclin-dependent kinase 4 and 6 inhibitor*':ab,ti OR 'cyclin-dependent kinase 4/6 inhibitor*':ab,ti OR 'cdk4 and cdk6 inhibitor*':ab,ti OR 'cdk4/6 inhibitor*':ab,ti OR 'cdk inhibitor*':ab,ti OR 'cdki':ab,ti

#5 'palbociclib':ab,ti OR 'ribociclib':ab,ti OR 'abemaciclib':ab,ti

#6 #4 OR #5

#7 #3 AND #6

#8 'crossover procedure':de OR 'double-blind procedure':de OR 'randomized controlled trial':de OR 'single-blind procedure':de OR random*:de,ab,ti OR factorial*:de,ab,ti OR crossover*:de,ab,ti OR ((cross NEXT/1 over*):de,ab,ti) OR placebo*:de,ab,ti OR ((doubl* NEAR/1 blind*):de,ab,ti) OR ((singl* NEAR/1 blind*):de,ab,ti) OR assign*:de,ab,ti OR allocat*:de,ab,ti OR volunteer*:de,ab,ti

#9 #7 AND #8

WEB OF SCIENCE

#1 TS=(breast OR mammary) AND TS=(carcinoma OR neoplasm OR tumor OR cancer) AND TS=(metastatic OR advanced OR metastases OR metastasis)

#2 TS=('cyclin-dependent kinase 4 and 6 inhibitor*' OR 'cyclin-dependent kinase 4/6 inhibitor*' OR 'CDK4 and CDK6 inhibitor*' OR 'CDK4/6 inhibitor*' OR 'CDK inhibitor*' OR 'CDKi')

#3 TS=(palbociclib OR ribociclib OR abemaciclib)

#4 #2 OR #3

#5 #1 AND #4

#6 TS= clinical trial* OR TS=research design OR TS=comparative stud* OR TS=evaluation stud* OR TS=controlled trial* OR TS=follow-up stud* OR TS=prospective stud* OR TS=random* OR TS=placebo* OR TS=(single blind*) OR TS=(double blind*)

#7 #5 AND #6

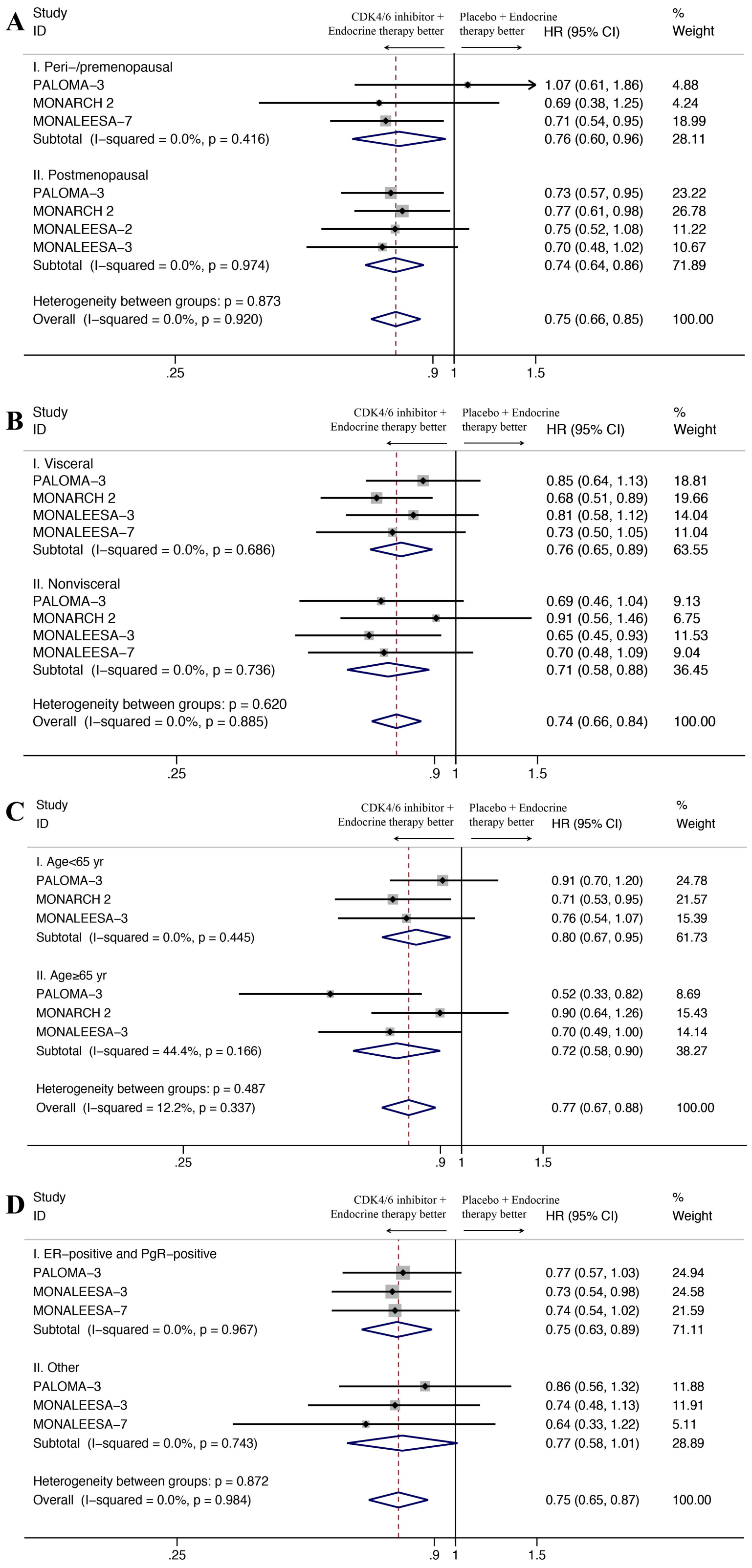

Figure S1 Forest plot showing pooled hazard ratios of overall survival in subgroup stratified by menopausal status (A), visceral involvement (B), age (C), and hormone-receptor status (D).

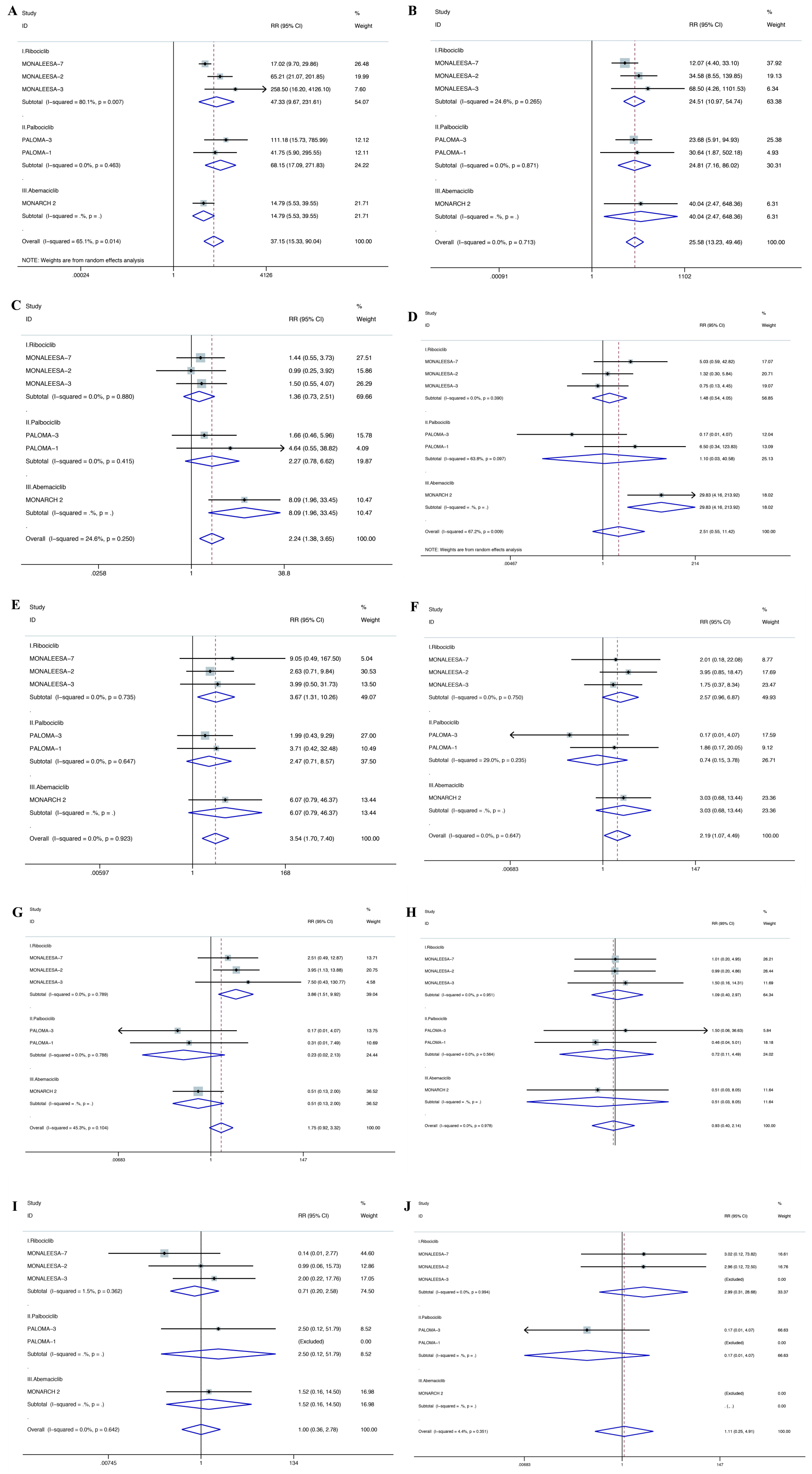

Figure S2 Forest plot showing pooled risk ratios in subgroup stratified by different CDK4/6 inhibitors of grade 3-4 neutropenia (A), leucopenia (B), anaemia (C), diarrhea (D), fatigue (E), nausea (F), vomiting (G), arthralgia (H), headache (I) and hot flush (G).

sTable1 Risk of bias assessment of the included randomized controlled trials using the Cochrane Collaboration's Tool

Study	Random sequence generation	Allocation concealment	Blinding of participants and personnel	Blinding of outcome assessment	Incomplete outcome data	Selective outcome reporting
PALOMA-1	Low risk	Low risk	High risk	Unclear	Low risk	Low risk
PALOMA-3	Low risk	Low risk	Low risk	Low risk	Low risk	Low risk
MONARCH 2	Low risk	Low risk	Low risk	Low risk	Low risk	Low risk
MONALEESA-2	Unclear	Unclear	Low risk	Low risk	Low risk	Low risk
MONALEESA-3	Unclear	Unclear	Low risk	Low risk	Low risk	Low risk
MONALEESA-7	Low risk	Low risk	Low risk	Low risk	Low risk	Low risk
